# Automatic quantification of left atrium volume for cardiac rhythm analysis leveraging 3D residual UNet for time-varying segmentation of ECG-gated CT

**DOI:** 10.1016/j.csbj.2025.04.039

**Published:** 2025-05-13

**Authors:** Rossana Buongiorno, Ilaria Verdirame, Francesca Dell'Agnello, Benigno Marco Fanni, Katia Capellini, Alberto Clemente, Vincenzo Positano, Sergio Berti, Simona Celi

**Affiliations:** a BioCardioLab, Bioengineering Unit, Fondazione Toscana G. Monasterio, Massa, 54100, Italy; b Department of Information Engineering, University of Pisa, via G. Caruso, Pisa, 56122, Italy; c Department of Radiology, Fondazione Toscana G. Monasterio, Pisa, 56122, Italy; d Adult Cardiology Unit, Fondazione Toscana G. Monasterio, Massa, 54100, Italy

**Keywords:** Left atrium, Segmentation, Time varying ECG-gated CT, Volume variation analysis, Atrial fibrillation

## Abstract

Atrial fibrillation (AF) is a heart condition widely recognized as a significant risk factor for stroke. Left atrial (LA) volume variation has been identified as a key predictor of AF, and several researchers have proposed deep learning models capable of quickly providing this measurement by processing computed tomography (CT) or magnetic resonance images. In clinical imaging, time-varying ECG-gated CT offers precise information about LA anatomy and function, which could help in developing personalized treatment plans for AF patients. Furthermore, advancements in time-varying dataset acquisition indicate the potential for expanding the role of CT in the management of AF patients through specialized processing techniques. However, automatic segmentation of the LA across all cardiac phases remains challenging due to significant variations in both anatomical structures and image signals throughout the cardiac cycle. To overcome these challenges, this study presents a comprehensive AI-based framework designed to segment the LA across the entire cardiac cycle and classify patients with AF. Specifically, our framework employs a customized Residual 3D-UNet model to segment the LA from time-varying ECG-gated CT scans and utilizes a One-Class Support Vector Machine (OCSVM) to distinguish patients in sinus rhythm (SR) from those with AF. A dataset of 93 time-varying ECG-gated CT scans was retrospectively collected: 60 patients were used for the segmentation task, while 33 patients were used for the classification task. The Residual 3D-UNet model demonstrated high accuracy, achieving a mean Dice score of 0.94, with consistent precision (94.45%) and recall (94.83%) across ten cardiac phases. The OCSVM achieved 78.7% accuracy with high specificity (86.3%), effectively minimizing the risk of misclassifying AF as SR, although sensitivity was lower at 70%, demonstrating the potential of automated segmentation and rhythm classification, providing a potential valuable tool for AF diagnosis.

## Graphical abstract

**Figure:**
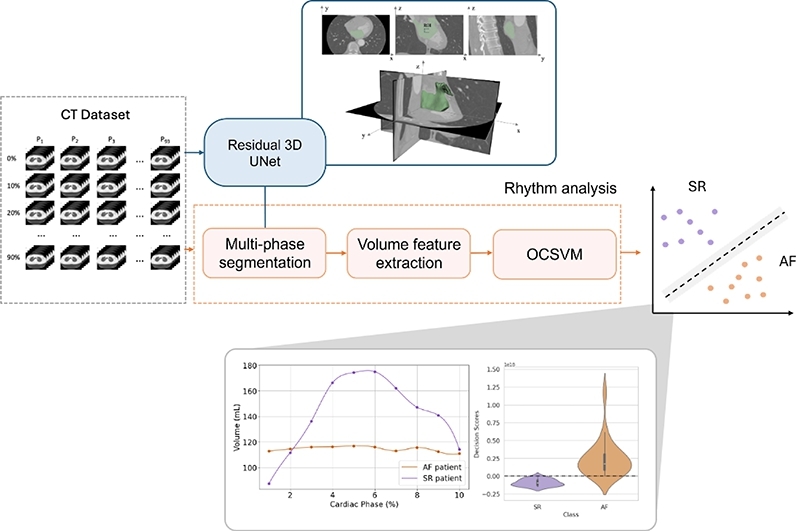

## 1 Introduction

Atrial fibrillation (AF) is a cardiac arrhythmia that disrupts the normal sinus rhythm (SR) of the heart [1]. Patients with chronic AF often experience remodeling of the left atrial appendage (LAA), characterized by reduced contractility, slower blood flow velocity, and an increased probability of clot formation due to blood stasis within the appendage [2]. These changes significantly affect the morphology and functional activity of the left atrium (LA), thus elevating the risk of stroke [3]. To mitigate this risk, the LAA occlusion (LAAO) procedure involves deploying a self-expanding device at the opening of the LAA, to reduce the potential for clot formation.

Over the past decade, computed tomography (CT) has proven to be a valuable diagnostic tool for both the planning of the LAAO procedure [4] and the monitoring of its outcomes during follow-up [5]. Particularly, ECG-gated CT provides 3D imaging of the heart, capturing detailed anatomical information throughout the entire cardiac cycle. To effectively plan the LAAO procedure, morphological analysis using ECG-gated CT images offers precise measurements of the LAA ostium diameter and its shape, the ostium relative position to the fossa ovalis, and the presence of pre-existing thrombi within the LAA [6]. In contrast, follow-up acquisitions provide information about the device position and potential peri-device leaks. In terms of functional assessment, ECG-gated CT offers dynamic information on the kinematics of cardiac structures, both under normal SR and in cases of AF, as AF impacts the dynamics and hemodynamics of the LA [7]. Patients in SR typically show normal contractility and stable flow dynamics, whereas those with AF experience disorganized contractions and irregular, often turbulent, flow patterns that increase the risk of thrombus formation. As a result, understanding cardiac rhythm is crucial for assessing stroke risk, tailoring interventions, and guiding post-procedural monitoring and treatment. AF, in particular, is linked to a higher risk of thromboembolic events, requiring more intensive follow-up and potentially influencing long-term management strategies.

While the ECG signal works in terms of electrical data and cannot capture structural abnormalities or changes in atrial dynamics during the cardiac cycle, its integration with CT imaging allows for a more comprehensive assessment by providing both anatomical and functional insights. Specifically, the ECG signal associated with CT images enables the precise identification of areas with greater or lesser contraction [8]. CT imaging provides valuable information on atrial enlargement, wall thinning, and fibrosis. These structural markers could support early risk stratification and personalized management, particularly in patients with paroxysmal AF [9]. Thus, the combination of anatomical and functional data helps mapping the structural alterations of the regions with contractile dysfunction involved in rhythm disturbances, enhancing the overall analysis of cardiac function [10]. This could provide deeper insights into the mechanisms underlying AF, facilitating improved risk stratification for stroke and optimizing treatment strategies tailored to individual patients.

Looking ahead, we aim to further refine this approach by mapping structural variations in the left atrium and left atrial appendage to identify specific regions involved in rhythm disturbances. On the one hand, acquiring multiple volumetric datasets at different phases of the cardiac cycle enables precise observation of structural dynamics. On the other hand, processing these datasets can be highly demanding, particularly when retrospective reconstructions are performed every 5–10% of the cardiac cycle, resulting in up to 20 volumetric datasets per subject. Calculating volumes at each phase is especially challenging, as it requires a segmentation process that, when manually performed, is time-intensive, costly in routine clinical practice, and prone to intra- and inter-operator variability due to the complex anatomy and patient-specific differences. This variability can lead to over- or underestimation of measurements, potentially compromising the accuracy of the analysis. Additionally, CT acquisition generates a 3D dataset, which inherently makes its processing time-consuming.

To address these challenges, a significant amount of effort in the literature has been dedicated to the automatic 3D segmentation of cardiac and cardiovascular structures by developing Artificial Intelligence (AI) methods, particularly through Machine Learning (ML) and Deep Learning (DL) approaches, such as Convolutional Neural Networks (CNNs) [11], [12]. Recent advancements in DL have significantly improved the segmentation of the LA and LAA in CT, TEE and magnetic resonance imaging (MRI), primarily driven by the increasing use of state-of-the-art approaches like fully convolutional networks (FCNs) [13], [14], U-Net architectures [15], [16], [17], and their modifications, such as 3D-UNets, both single [18], [19], [20] and multi-view [21], [22], and attention-based mechanisms, to capture both spatial and contextual information from 3D medical scans [23], [24], [25], [26]. Networks with residual connections have also emerged as a powerful addition to segmentation models, preserving important features across the layers and enhances the ability to detect complex structures of LA and LAA [27], [28]. Additionally, multi-task learning frameworks and hybrid models combining CNNs with other machine learning techniques have been proposed to enforce anatomical consistency in segmentation results [29], [30], [31]. Instead, in situations where annotated data is limited, transfer learning has improved segmentation performance leveraging models pre-trained on large datasets and fine-tuning them for specific tasks [32], [33]. Despite advancements, achieving robust and generalizable LA segmentation remains challenging, especially when accurate segmentation is required across all the cardiac phases. Notably, DL has yet to be widely applied for segmenting the entire cardiac cycle in ECG-gated CT images, which is essential for tracking heart motion and accurately estimating volume variation over time. The complex and variable shape of the LAA throughout the cycle, along with noise and imaging artifacts, further complicates segmentation. Additionally, the scarcity of large annotated datasets poses a significant challenge, necessitating tailored training approaches. Consequently, most existing studies focus on MRI-based segmentation rather than CT or CT angiography, which are the primary imaging tools for LAA occlusion planning. Given that CT scans uniquely capture the dynamic anatomy of the LA, there is a pressing need for focused research to enhance segmentation accuracy across the cardiac cycle, ensuring reliable volume estimation for improved clinical decision-making.

Furthermore, so far, no studies have yet used ML techniques to characterize SR or AF based on imaging features extracted from the segmentation of LA volumes across the cardiac cycle in ECG-gated CT scans. While previous research has applied ML methods to classify arrhythmias based on intracardiac electrical patterns during AF, using time-series signals [34], [35], [36], [37] or time-frequency scalograms [38], other studies have modeled cardiac wall deformation to predict AF recurrence by analyzing shape features and clinical factors [39]. However, most of this work focused on LA shape differences in CT scans between AF patients with and without post-ablation recurrence, rather than supporting the planning of LAAO procedures. Developing such methods could bridge the gap between anatomical segmentation and clinical rhythm assessment, improving diagnostic accuracy and supporting clinical decision-making.

In this study, we developed a comprehensive AI-based framework for performing 3D segmentation of the LA across ten phases of the cardiac cycle using ECG-gated CT. From these 3D segmentations, we extracted key clinical features, including LAEI, LAEF, and AP. These features were then used to classify cardiac rhythms and differentiate SR from AF. To automate this classification, we employed a One-Class Support Vector Machine (OCSVM) model, evaluating the efficacy of a fully image-based pipeline for rhythm classification. The segmentation process was carried out using a supervised training approach, while an unsupervised method was applied to classify the morphological features associated with cardiac rhythm. The paper is organized as follows: Section 2 details the dataset used and the methodology employed to design, train, and test the Residual 3D-UNet, as well as to validate the OCSVM model using the image-based features extracted from the resulting segmented volumes. Then, in Section 3, we present the results, and finally, Section 4 provides a discussion and conclusion of the paper.

## 2 Material and methods

**Fig. 1:**
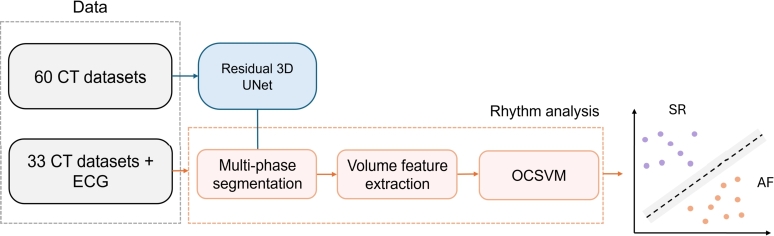
Diagram illustrating the workflow followed for this study.

In this section, we provide an overview of the data and methods, schematically summarized in Fig. 1. Details on the datasets used for training and validating the segmentation and classification models, along with the ground truth delineation process, are presented in Section 2.1. Section 2.2 details the preprocessing steps and augmentation procedures. Section 2.3 focuses on the Residual 3D-UNet, specifically tailored for this task, covering the model architecture, k-fold cross-validation scheme, and other implementation specifications. Section 2.4 outlines the methodology used to develop the OCSVM algorithm for distinguishing patients in SR from those with AF. Section 2.5 describes the metrics used to assess the model performances. Finally, section 2.6 describes the comparative analysis of OCSVM with other five binary unsupervised classification methods.

### 2.1 Data collection and labeling

**Fig. 2:**
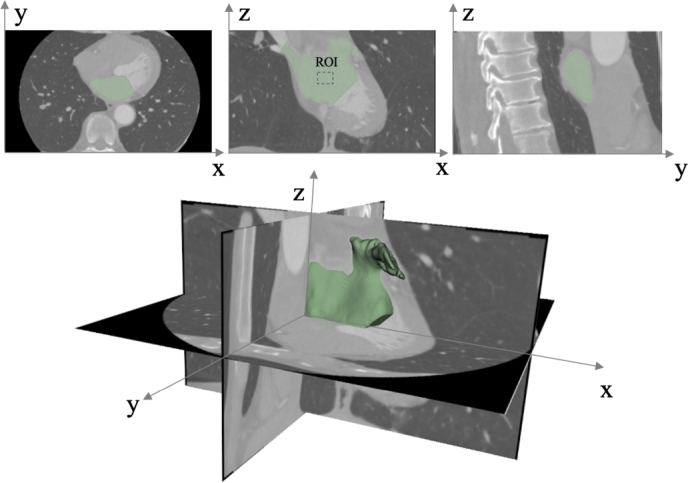
Ground truth manually segmented in the axial (x-y), coronal (x-z), and sagittal (y-z) planes of a CT scan and the corresponding 3D visualization. The ROI used for the SNR calculation is also shown in the coronal view.

*Imaging protocols* - All CT scans were acquired in accordance with a standard clinical protocol [4], by using a 320-detector scanner (Toshiba AQUILION One scanner), and retrospective ECG-gating, covering the 0–90% of the RR interval at 10% increments (10 frames). Adjacent 512×512 axial images were reconstructed using the FC43 convolution kernel. The tube voltage was set to 100 kVp, with a tube current of 200 mA and an exposure time of 350 ms. The mean radiation dose was 16.7 mGy. All scans were performed following intravenous administration of iodinated contrast agent. Table 1 reports the main data characteristics, expressed as mean values and standard deviations (SD) of the number of slices, pixel dimensions, and slice dimensions, are reported, along with the Signal to Noise Ratio (SNR). To calculate the SNR, we defined both the signal and the noise. The signal was the mean intensity of the pixel values included in a ROI within the LA (Fig. 2). The noise was the SD of the pixel values included in a ROI of the image where no signal was present, i.e., the background. Once obtained both measurements, we computed the SNR as the ratio between the mean signal intensity (*S*) and the SD (*σ*) of the noise as follows:

(1) $$\text{SNR}=\frac{S}{\sigma }$$

**Table 1:** Dataset characteristics.

Characteristics	Mean ± SD
Number of slices	142 ± 14
Pixel spacing (mm)	0.45 ± 0.4
Slice thickness (mm)	1 ± 0
SNR	0.79 ± 0.09

*Patient cohort criteria* - 60 ECG-gated cardiac CT datasets were retrospectively collected from our patient database to train and validate the Residual 3D-UNet. Inclusion criteria were the diagnosis of non-valvular AF, moderate to high risk for thrombus formation, based on established clinical scoring systems (e.g., CHA_2_DS_2_-VASc), and unsuitable for long-term oral anticoagulation therapy due to contraindications such as high bleeding risk or intolerance [40], [41]. Specifically, AF rhythm was classified when the R-R interval fell outside the range of 654.6-1141.4 ms [42]. Exclusion criteria included the presence of significant valvular disease, prior LAAO procedures, or incomplete CT data.

Each CT dataset corresponded to a different patient diagnosed with non-valvular AF. The final cohort comprised 35 males and 25 females, with a mean age of 76 and 73 years, respectively. The AF subtypes included were paroxysmal (12 males and 10 females), persistent (6 males and 6 females), and permanent (17 males and 9 females), and the heart rate distribution had a mean value of 103 ± 19 bpm. In addition, a dataset of 33 patients with similar characteristics, provided with the clinical rhythm evaluations performed by expert cardiologists, was used to train and validate the OCSVM algorithm for rhythm classification. Among these, 22 were in AF state.

*Data labeling* - To ensure the quality and consistency of the annotations, the ground truths were established as an agreement between three experts: two with more than six years of experience and one with one year of experience. An annotation protocol was developed prior to the start of the process, including detailed guidelines on the anatomical structures of interest, boundary definitions, and handling of ambiguous regions. These guidelines were shared among annotators to promote label consistency, and primarily involved manual segmentation, performed using 3D Slicer (www.slicer.org). The manual segmentation consisted of a threshold-based segmentation, followed by a clipping process to remove undesired high-signal areas such as bones, the aorta, and the left ventricle [43]. Finally, the plane of the mitral valve was established as the boundary between the left atrium and the left ventricle, while the pulmonary veins were cut using a plane positioned prior to their bifurcation (Fig. 2). Furthermore, we conducted a variability analysis on a representative subset of 10 dataset to asses inter- and intra-observer agreement [44]. Specifically, the two expert annotators independently segmented the subset and the Dice Score was computed to quantify segmentation overlap. For intra-observer variability, each annotator re-segmented the subset after a 2 weeks interval. The mean Dice score obtained in both inter- and intra-observer comparisons were used as a proportional measure of annotation consistency, equal to 0.97 ±0.02 and 0.99 ±0.004, respectively.

### 2.2 Data preprocessing

*Data preparation -* After collection, the data underwent a conversion process into the NIfTI format, chosen for its structural simplicity, which proves beneficial for handling 3D volumetric scans. To improve efficiency and speed-up model convergence, the NIfTI-converted data underwent preprocessing operations, including clipping and normalization of greyscale values. The clipping operation consisted of restricting the Hounsfield unit (HU) values to the specific range of [-350 HU; 800 HU] to cover the spectrum of greyscale levels from lung tissue to bone, including the contrast agent-enhanced signal in the circulatory system. This preprocessing step allows the model to focus on the relevant grayscale values, setting the HU values above +800 HU to +800 HU and those below -350 HU to -350 HU. After clipping, the min-max normalization procedure adjusted the resulting HU values to align within the standardized range of [0, 1]. The normalized volumes were then cropped and resampled to ensure homogeneity. Specifically, volumes were cropped within the segmented regions, and resampled to standardize voxels dimensions to 1×1×1 mm^3^. For data augmentation, we applied three online affine transformations to the preprocessed volumes: rotation, shearing, and scaling. Random rotations were performed within small distortion intervals, with angles of ±0.2 radians across the *x*-*y*, *x*-*z*, and *y*-*z* planes. Shearing altered the NIfTI volumes along the *x*, *y*, and *z* axes (see Fig. 2), with the shearing matrix preserving diagonal elements while distorting off-diagonal elements across different axis combinations. The range of the shearing operation was randomly varied within ±0.05 units. Lastly, scaling introduced a random 15% variation in volume size. This augmentation strategy expanded the dataset while ensuring that the augmented data closely resembled the original dataset, preserving consistency across patients in similar positions. The shearing transformation skews the shape of the NIfTI volumes along the *x*, *y*, and *z* axes. To apply this augmentation technique, the matrix *S* maintains each diagonal element unchanged, while off-diagonal elements undergo distortion across various axis combinations:

(2) $$\begin{array}{c} S=\left[\begin{array}{cccc} 1 & s_{x} & s_{x}\cdot s_{y} & 0\\ s_{y} & 1 & s_{y}\cdot s_{z} & 0\\ s_{z} & s_{z}\cdot s_{x} & 1 & 0\\ 0 & 0 & 0 & 1 \end{array}\right] \end{array}$$

$s_{x}$, $s_{y}$, and $s_{z}$ refer to the distortions along the three axes respectively.

*Data splitting -* As mentioned above, one of the primary objectives was to train a model capable of accurately segmenting each phase of the cardiac cycle, corresponding to different degrees of atrial and ventricular dilation, while maintaining consistent performance. To achieve this, we structured the preprocessed data into a rigorous 5-fold cross-validation framework. The entire dataset was randomly divided at the patient level into 36 for the training set, 12 for the validation set, and 12 for the test set in each fold. This allocation ensured that during validation, the model's predictions were based on unseen data from the training phase, effectively evaluating its generalization capability. Furthermore, in each fold of our methodology, the model was systematically trained on six distinct phases of the cardiac cycle from the patients in the training set, validated on two phases from the patients in the validation set, and rigorously tested on the remaining two phases from the patients in the test set, as detailed in Table 2. More precisely, the test set in each fold encompassed a total of 12 patients: six from one phase of the cardiac cycle and six from a phase placed 50% on the total cycle duration apart (for example, phases 0%-50% in the first fold, 10%-60% in the second fold, and so forth).

**Table 2:** Phases of the cardiac cycle included in the training, validation, and test sets for each fold.

Fold	Training	Validation	Test
1	10%-20%-30%-60%-70%-80%	40%-90%	0%-50%
2	20%-30%-40%-70%-80%-90%	0%-50%	10%-60%
3	0%-30%-40%-50%-80%-90%	10%-60%	20%-70%
4	0%-10%-40%-50%-60%-90%	20%-70%	30%-80%
5	0%-10%-20%-50%-60%-70%	30%-80%	40%-90%

By employing this structured approach, we aimed to ensure that the average performance metrics calculated across all test sets in every fold provided a comprehensive and unbiased assessment of the model's efficacy across all phases of the cardiac cycle. After cross-validation, the best-performing model was selected for calculating the volumetric features.

### 2.3 Residual 3D-UNet for LA segmentation

The following paragraphs include the details about the Residual 3D-UNet tailored in this work in terms of the architecture (Section 2.3.1), and training and validation setup (Section 2.3.2).

#### 2.3.1 Model architecture

**Fig. 3:**
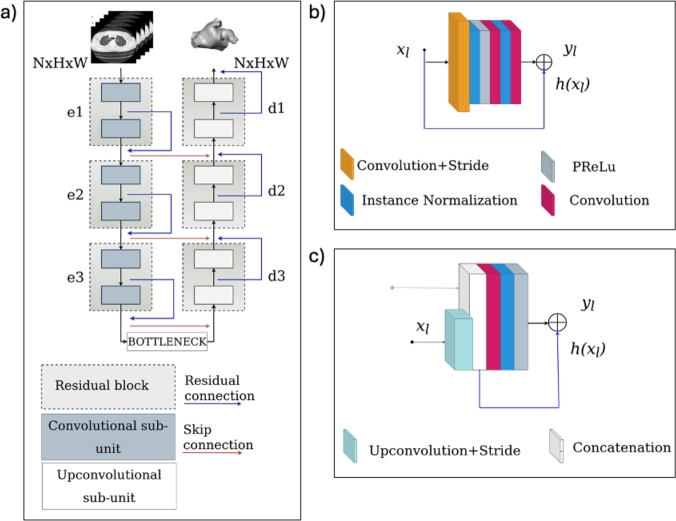
(a) Model architecture; (b) convolutional residual sub-unit and (c) up-convolutional residual sub-unit. e1, e2, and e3 refer to the encoding stages, while d1, d2, and d3 indicate the decoding stages.

The selected architecture for the current task was a 3D-UNet with residual blocks containing two residual sub-units at each stage of both the encoder and the decoder (see Fig. 3a). The depth of both the encoder and the decoder path was set equal to 3. The input layer processed volumetric CT scans of patients, represented as stacks of gray-scale 2D images with dimensions *N*×*R*×*C*, where *N* denotes the number of slices per CT scan, which varies across patients, and *R*×*C* represents the dimension of the images, equal to 512×512 pixels each, and consistent for all patients. The number of feature maps doubled across each residual block along the down-sampling path, ranging from 32 to 256 maps. In contrast, along the decoding path, starting from the 256 maps, each residual block halved the number of feature maps. Additionally, skip connections were put at each stage to restore spatial information lost during down-sampling. This was achieved by concatenating the encoder's feature maps with their corresponding decoded features. Following concatenation, the resultant feature maps underwent three successive 3×3 residual blocks. The sigmoid activation function was used for the last convolutional layer to map each resulting feature vector to the desired number of classes, thus returning a *N*×*R*×*C* map as the binary volume comprising the left atrium and appendage, and with the same size of the input. As mentioned above, each stage of the encoding and decoding paths consisted of a residual block with two sub-units (Fig. 3b). To increase the ability of the model to integrate contextual information, residual connections were added to each residual block, in order to recursively process the input only once at each stage. The residual blocks and their functioning are described in detail in the following paragraph.

*Residual block -* The input of the first sub-units in each residual block was the output of the preceding residual block except for the first convolutional one, i.e., *e*1, which took as input the stacks of *N* gray-scale 2D images with 512×512 pixels corresponding to the volumetric CT scans as described above. The operations performed by each convolutional residual sub-unit initially consisted of a strided 3×3 convolution, followed by an instance normalization and a Parametric Rectified Linear Unit (PReLU) activation (Fig. 3b). After the PReLU activation layer, each block includes two sequential convolution operations, interleaved with steps of instance normalization. The up-convolutional residual sub-units (Fig. 3c) consisted of a strided up-convolution, followed by a convolutional, and instance normalization, and a PReLu activation layer. In both cases, instance normalization was applied to decrease the impact of contrast shifting thus ensuring a consistent input image contrast without distortion from batches of images characterized by different contrast ranges [45]. PReLU layers were used to help the network improve its activation learning, thereby boosting segmentation performance [46]. Finally, the output of the first sub-units of each residual block was also concatenated with the output of the second sub-units via residual connections thus allowing the addition of the identity mapping, i.e., adding the input to the output. The mathematical explanation of the residual blocks is described by the following equations [47]:

(3) $$y_{l}=h\left(x_{l}\right)+F\left(x_{l},W_{l}\right)$$

(4) $$x_{l+1}=f\left(y_{l}\right)$$

where $x_{l}$ and $y_{l}$ are the input and the output features to the $l_{th}$ residual sub-unit, respectively. $W_{l}=\left\{{W_{l,k}|}_{1\leq k\leq K}\right\}$ is a set of weights and biases associated with the $l_{th}$ residual sub-unit, and *K* is the number of layers in a residual sub-unit. We set *K* equal to 2 in the convolutional residual sub-units and 1 in the upconvolutional residual sub-units. F denotes the operations performed by convolutional and up-convolutional residual sub-units. The function *f* is the operation after element-wise addition, and the function *h* an identity mapping implemented by the residual connections. In this work, *f* is also an identity mapping, so we obtain:

(5) $$x_{i+1}=y_{i}=x_{i}+F\left(x_{i},W_{i}\right)$$

Generally, the feature of any deep unit is the summation of the outputs of all preceding residual functions and the information flow in the network is improved. In fact, residual connections are generally used to iteratively refine predictions reusing the features from earlier steps, aiding in the learning of hierarchical representations.

#### 2.3.2 Training and validation setup

We selected the Dice loss (DL) as loss function due to its effectiveness in handling classification tasks with unbalanced data. Specifically, the LA, which are the areas of interest to be segmented, always constitute a minority portion of each entire CT scan. Additionally, the homogeneous appearance of the region of interest, influenced by the high signal intensity due to the diffusion of the contrast agent, makes the Dice score particularly suitable as this metric assesses the alignment between predictions and ground truth. In particular, the implemented DL, for the discrimination of two classes (LA and background) can be expressed as [48]:

(6) $${\mathrm{DL}}_{2}=1-\frac{\sum_{n=1}^{N}p_{n}r_{n}+\epsilon }{\sum_{n=1}^{N}(p_{n}+r_{n})+\epsilon }-\frac{\sum_{n=1}^{N}\left(1-p_{n}\right)\left(1-r_{n}\right)+\epsilon }{\sum_{n=1}^{N}(2-p_{n}-r_{n})+\epsilon }$$

where $p_{n}$ represents the value of the $n_{th}$ pixel in the predictions volumes P, and $r_{n}$ represents the values of the $n_{th}$ pixel in the reference volume R. *ϵ* is used to ensure the loss function stability by avoiding the numerical issue of dividing by 0, namely when R and P are empty.

An augmentation factor equal to 4 was applied. Adam Optimizer was used as optimization algorithm, with fixed a learning rate equal to 0.0001, the exponential decay rates for the moving average of the gradient equal to 0.9, and the squared gradient equal to 0.999.

The training ran on Pytorch (version 2.2.0) and was coded in Python 3.8.18. All experiments were performed under a Ubuntu 22.04.3 LTS operative system. We set the number of epochs to 500 and saved the trained models at each epoch to evaluate their performance later. Early stopping was implemented by monitoring the validation loss after each epoch, with a patience of 20 epochs, indicating the maximum number of consecutive epochs without improvement before stopping the training. For each model, the weights were saved at every epoch, and testing was conducted using the weights corresponding to the epoch with the lowest validation loss within the 20-epoch patience window.

In order to make the training independent from the data split, we performed a 5-fold cross-validation as described in Section 2.2.

*Parameter inizialitation* - The Residual 3D-UNet was implemented using the MONAI framework. Model weights were initialized using default initialization methods, which typically apply Kaiming initialization to convolutional layers. A fixed random seed was set to ensure reproducibility across runs. This affected all stochastic elements of the pipeline, including weight initialization, data augmentation, and data shuffling. No pretrained weights were used, and the training was performed entirely from scratch.

### 2.4 Rhythm analysis

After being trained and validated, the OCSVM model was applied to distinguish between SR and AF rhythms, processing specific volumetric parameters such as the Left Atrial Expansion Index (LAEI), Left Atrial Emptying Fraction (LAEF), and the Antero-Posterior diameter (AP). The features are presented in Section 2.4.1, while the OCSVM model in Section 2.4.2.

#### 2.4.1 Volumetric features extraction

We employed the best model from the 5-fold cross-validation, as described in Section 2.2, to segment the 10 phases of 33 CT dataset from a separate dataset specifically collected for the classification task. First, for each cardiac phase, the ten LA volumes (LA${}_{Vi}$) were computed. The LA${}_{Vi}$ for each patient was obtained by summing the segmented voxels and multiplying by the voxel dimensions (*dx* and *dy*, as described in the pixel spacing measures in Table 1, and *dz*, as noted in the slice thickness values). Then, the maximum (LA${}_{Vmax}$) and minimum volume (LA${}_{Vmin}$) for each patient were identified, followed by the calculation of the volumetric features.

The LAEI quantifies the LA capacity to expand during diastole, thus reflecting the compliance and reservoir function:

(7) $$\text{LAEI}=\left(\frac{\text{LA}{}_{Vmax}-\text{LA}{}_{Vmin}}{\text{LA}{}_{Vmin}}\right)\times 100$$

The LAEF represents the proportion of blood emptied from the LA during atrial contraction. It is defined as the percentage decrease in LA volume from the maximum volume at end-diastole to the minimum volume at end-systole:

(8) $$\text{LAEF}=\left(\frac{\text{LA}{}_{Vmax}-\text{LA}{}_{Vmin}}{\text{LA}{}_{Vmax}}\right)\times 100$$

**Fig. 4:**
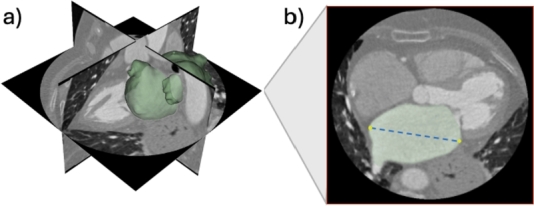
(a) 3D model and (b) LA antero-posterior diameter obtained from the segmentation of the LA by the trained Residual 3D-UNet.

The AP is the measurement of the LA size along the antero-posterior axis. According to [49], [50], this diameter is measured at the end of ventricular systole, when the atrium is at its maximal volume. At this phase, the slice with the largest surface area was selected to extract the LA border, and the diameter was determined as the maximum distance between each border pixel and its neighboring pixels, to account for variations in atrial shape among patients (Fig. 4).

#### 2.4.2 OCSVM

An OCSVM model maps data into a higher-dimensional space using an implicit transformation function *ϕ* and seeks to learn a decision boundary that separates most of the data. A small fraction of points is left outside the boundary and classified as outliers. This approach enables the identification of data points that significantly deviate from the main distribution, labeling them as anomalies [51], [52]. Given that the distribution of the data showed a predominance of AF patients (22 out of 33 cases), in our specific case, the OCSVM algorithm treats SR patients as outliers. Thus, given a training dataset $X=\left\{x_{1},\ldots ,x_{n}\right\}$, where $x_{i}\in R^{d}$ (with d=3 corresponding to the number of feature types extracted, and *n* being the number of training samples, i.e., 33), the OCSVM algorithm maps the data to a higher-dimensional feature space using a kernel function *ϕ*. Next, a hyperplane that best separates the data from the origin with the maximum margin is found by solving the following quadratic programming problem:

(9) $$\arg\min_{w,\xi ,\rho }\frac{1}{2}{\left\|w\right\|}^{2}+\frac{1}{\nu n}\sum_{i=1}\xi_{i}-\rho ,$$

subject to the constraints:

(10) $$\langle w,\phi (x_{i})\rangle \geq \rho -\xi_{i},\;\xi_{i}\geq 0$$

where **w** is the normal vector, $\xi =[\xi_{1},\ldots ,\xi_{n}]$ represents the misclassification degrees for each data point, *ρ* is the offset of the desired hyperplane, and ν∈(0,1] is a trade-off parameter that controls the upper bound on the fraction of training samples that lie outside the decision boundary and the lower bound on the fraction of data points that must be retained to avoid over-simplifying the OCSVM model. Since the kernel function *ϕ* can take different forms, we employed a grid search approach by systematically varying the *ϕ* function and the values of *c*, *d*, *γ*, and *ν*. Specifically, we tested *ϕ* functions in polynomial ($\phi_{P}$), linear ($\phi_{L}$), Radial Basis Function ($\phi_{RBF}$), and sigmoid ($\phi_{S}$) forms:

(11) $$\phi_{P}(x_{i},x_{j})={\left(x_{i}^{T}x_{j}+c\right)}^{d}$$

(12) $$\phi_{L}(x_{i},x_{j})=x_{i}^{T}x_{j}$$

(13) $$\phi_{RBF}(x_{i},x_{j})=\exp(-\gamma ||x_{i}-x_{j}|{|}^{2})$$

(14) $$\phi_{S}(x_{i},x_{j})=\tanh(\gamma x_{i}^{T}x_{j}+r)$$

where *c* is a constant, *d* is the degree of the polynomial, *γ* is a parameter that controls the kernel steepness, and *r* is a constant that shifts the function.

*OCSVM tuning -* To identify the optimal hyperparameters for the OCSVM algorithm, we employed a grid search approach by systematically varying the *ϕ* function, and the values of *c*, *d*, *γ*, and *ν*. Specifically, we assigned values to c∈[0,0.3], d∈[2,5], and *γ* as follows:

(15) $$\gamma =\frac{1}{n\times \mathrm{var}(X)}$$

As *γ* increases, the number of support vectors decreases, leading to looser decision boundaries. Similarly, the parameter *ν* influences the shape of the decision boundaries. Higher values of *ν* result in an increased number of misclassified training samples [53]. Since *ν* is closely associated with the proportion of training samples positioned outside the decision boundary, it is typically set to a low value to maintain a low misclassification rate during training. For this reason, we set ν∈[0.05,0.2].

We employed the grid search algorithm with a custom decision scoring function, designed to measure the model performance based on the distances from the decision boundary. Formally, the decision function is given by:

(16) f(x)=〈w,x〉+b

where 〈w,x〉 denotes the dot product between the weight vector *w* and the point *x*. The vector *w*, which has dimension *d* when the data belong to a space $R^{d}$, determines the orientation of the hyperplane. The term *b* represents the bias and f(x) provides the signed distance of the point *x* from the hyperplane. The absolute distance from the point to the hyperplane is determined by the absolute value of the decision function f(x), which quantifies how far the point is from the hyperplane:

(17) dist(x)=|f(x)|=|〈w,x〉+b|

A small distance indicates that the point is near the hyperplane, suggesting that it lies close to the boundary between inlier and outlier data. Conversely, a larger absolute distance suggests that the point is farther from the hyperplane and is, therefore, more likely to be classified as an outlier.

Using this decision scoring function, we systematically evaluated different combinations of hyperparameters and selected the configuration that maximized the mean distance from the decision boundary, thus enhancing the model robustness in identifying the outliers (i.e., patients in SR). For each candidate set of hyperparameters, the function iteratively performed the following steps:

1. calculates the distances of each data point from the decision boundary;
2. applies the absolute value to ensure distances reflect the magnitude from the boundary, regardless of whether points are classified as positive or negative;
3. checks for invalid values to ensure that hyperparameter sets producing invalid scores are penalized during optimization;
4. returns the mean distance for valid cases, which serves as the scoring metric. A higher mean distance indicates a clearer separation between the normal and anomalous points.

*OCSVM validation -* After optimizing the OCSVM algorithm, we implemented a 5-fold cross-validation strategy designed to maintain non-overlapping groups. Each group appeared in the test set exactly once across all folds. The folds were structured to be approximately balanced, ensuring that each test set contained a comparable number of samples.

The 5-fold technique used was deterministic rather than random, with sample assignment based solely on the ordering of the groups. This approach ensured that all samples from the same group remained together in either the training or validation set. Regarding the distribution of patients across test folds, the first three folds included seven patients each, while the last two folds contained six patients each.

### 2.5 Evaluation metrics

To analyze the performance of the Residual 3D-UNet, we used metrics defined on the entire volume, such as 3D Dice score (*DS*), Precision (*Pr*) and Recall (*Re*), defined as follows:

(18) $$DS=\frac{2|P\cap GT|}{\left|P|+|GT\right|}$$

(19) $$Pr=\frac{\left|P\cap GT\right|}{\left|P\right|}$$

(20) $$Re=\frac{\left|P\cap GT\right|}{\left|GT\right|}$$

where *P* is the predicted segmentation and *GT* the ground truth. For evaluating the performance of the OCSVM model, we used *Sensitivity* to assess the model's ability to correctly identify negative instances, *Specificity* to measure how well the model detects positive instances, *Accuracy* (Acc) to evaluate the overall correctness of the model predictions, and *balanced accuracy* (B-Acc) to eventually address class imbalance, defined as:

(21) $$\text{Sensitivity}=\frac{TP}{TP+FN}$$

(22) $$\text{Specificity}=\frac{TN}{TN+FP}$$

(23) $$\text{Acc}=\frac{TP+TN}{TP+TN+FP+FN}$$

(24) $$\text{B-Acc}=\frac{\text{Specificity}+\text{Sensitivity}}{2}$$

where TP, TN, FP, and FN represent true positives, true negatives, false positives, and false negatives, respectively.

### 2.6 Comparison of classification methods

To investigate the effectiveness of the OCSVM in detecting SR rhythms in AF patients, we conducted a comparative analysis designing and optimizing five additional binary unsupervised classification methods. We followed the same approach as the OCSVM, optimizing the hyperparameters using a grid search algorithm, and setting up a five-fold cross-validation process to evaluate the performance of each method. We explored two strategies: the first involved using clustering techniques on the latent features extracted by an autoencoder specifically designed to process the LAEI, LAEF, and AP data [54]. The second approach applied clustering techniques directly to the LAEI, LAEF, and AP features [55].

*Autoencoder-based latent feature analysis with clustering -* The autoencoder compresses the input data into a lower-dimensional latent space to reconstruct the data back to its original form. This process allows to capture key patterns and characteristics of the data, minimizing the reconstruction error. Once the data is encoded into the latent space, we applied clustering techniques, such as Density-Based Spatial Clustering of Applications with Noise (DBSCAN) [56] and Gaussian Mixture Model (GMM) [57], to the latent features. In this way, we aimed to investigate the effectiveness of both a deterministic and a stochastic method to distinguish between SR and AF starting from the latent features.

*Direct clustering on volumetric features -* The second approach bypassed the use of an autoencoder and directly applied clustering techniques, such as DBSCAN, GMM, and Mean Shift (MS), to the volumetric features LAEI, LAEF, and AP, without performing any dimensionality reduction or feature extraction. By clustering directly on the volumetric features, we test whether the original feature set itself contained sufficient information for accurate rhythm classification. In addition to the aforementioned DBSCAN and GMM methods, we also applied the MS algorithm [58] directly on the features.

## 3 Results

### 3.1 LA segmentation

**Fig. 5:**
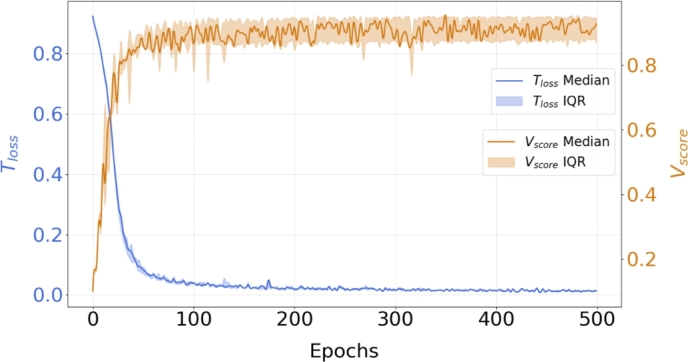
Training loss (*T*_*loss*_) and validation score (*V*_*score*_) trends over 500 epochs. The bands represent the relative Interquartile Range (IQR) of the two metrics.

Fig. 5 depicts the median training loss and validation accuracy across the folds over 500 epochs. The solid blue line indicates that the loss quickly drops, stabilizing around 0.1 after approximately 50 epochs. The narrow shaded blue region, representing the relative Interquartile Range (IQR) band, throughout most of the epochs, suggests consistent performance across the folds, with minimal variation. The validation accuracy (orange line) shows a rapid increase in the early epochs, but exhibits more fluctuations than the training loss.

As a consequence, the IQR band is wider than that of the training loss, although it still indicates relatively low variability, confirming that the model generalizes well across the different validation folds. Overall, the validation Dice score remains consistent throughout the remaining epochs, demonstrating that the model maintains its performance even as training continues. Table 3 shows the minimum values of the training loss ($T_{loss}$) and the maximum values of the validation score ($V_{score}$), which represent the average values calculated within the training and validation sets for each fold of the cross-validation, over the epochs.

**Table 3:** Minimum values of the Dice metrics for the training loss, and maximum values of the validation score representing the average values calculated within the training and validation sets for each fold of the cross-validation, over the epochs.

	Fold 1	Fold 2	Fold 3	Fold 4	Fold 5
$T_{loss}$	0.010	0.008	0.006	0.007	0.009
Epoch	414	495	484	495	484
$V_{score}$	0.95	0.94	0.95	0.88	0.91
Epoch	476	352	488	498	488

The consistency of model performance during training is supported by the minimal variation of the $T_{loss}$ values, which fall in the range between 0.006 and 0.010 across different folds. The $V_{score}$ range from 0.88 (reached during Fold 4) to 0.95 (during Fold 1), although Fold 4 exhibits slightly lower performance. The number of epochs varies, with Folds 2 and 4 reaching the maximum of 495 epochs, while Fold 1 stops earlier at 414 epochs.

Concerning the quantitative evaluation of the test sets of each fold, the numerical values obtained are reported in Table 4. As observed, the Residual 3D-UNet model performance remains generally stable across different folds, with only minor variations. The Dice score and Precision achieved their highest values of 0.96 ± 0.81 and 95.61% ± 2.57%, respectively, at fold 5. Meanwhile, the Recall reached its peak value of 96.79% ± 2.01% at fold 4. On average across all folds, the Dice score achieved a mean value of 0.95 ± 0.6, with an average Precision of 94.45% ± 1.11%, and a Recall of 94.83% ± 1.29%.

**Table 4:** Mean values and standard deviations of Dice score (DS), Precision (Pr), and Recall (Re) calculated on the test sets of each fold and across all folds. The highest values for each metric are highlighted in bold.

	Fold 1	Fold 2	Fold 3	Fold 4	Fold 5	ALL
DS	0.93 ± 0.22	0.95 ± 0.11	0.95 ± 0.13	0.95 ± 0.80	**0.96** ± **0.81**	0.94 ± 0.41
Pr	93.96% ± 3.69%	94.37% ± 2.51%	95.43% ± 2.53%	92.90% ± 4.27%	**95.61%** ± **2.57%**	94.45% ± 3.11%
Re	93.77% ± 3.36%	93.66% ± 6.70%	94.63% ± 4.78%	**96.79%** ± **2.01%**	95.32% ± 4.53%	94.83% ± 4.28%

**Fig. 6:**
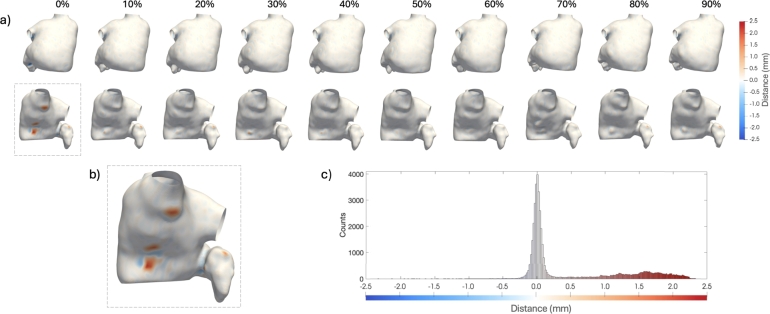
(a) An example of the distances between manual and automatic segmentation for all phases of the cardiac cycle, shown in a double view; (b) magnification of the worst case (phase 0%). The negative and positive values represent the node-to-node errors in terms of underestimation and overestimation of the AI model with respect to the manual one, respectively. The histogram (c) quantifies the number of nodes with the associated node-to-node error for phase 0%.

Fig. 6 presents an example illustrating the pointwise minimum distance between the ground truth and the predicted segmentations across all phases of the cardiac cycle for the patient with the highest segmentation error.

### 3.2 Rhythm analysis

Section 3.2.1 presents the results of volumetric feature extraction, starting from the calculation of segmented volumes. In Section 3.2.2, the OCSVM performance are reported.

#### 3.2.1 Volumetric features extraction

**Fig. 7:**
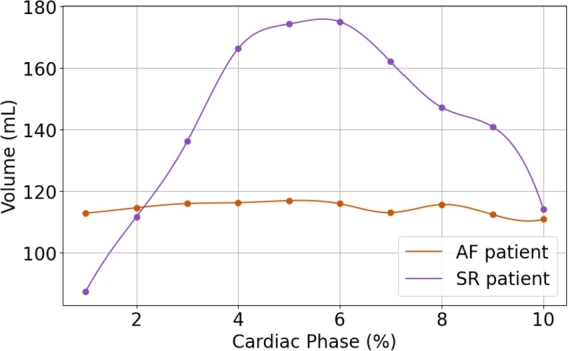
Representation of the cubic splines obtained from ten volumes across the cardiac cycle of a SR (violet) and AF (orange) patient.

Fig. 7 presents the dynamic changes in LA volume throughout the cardiac cycle for a patient in SR and AF. Worth noting that a significant difference in volume changes is observed between the two cases. The patient in SR maintains a volume change within a physiological range (90 mL), whereas the AF patient demonstrates a marked reduction in contractility.

**Fig. 8:**
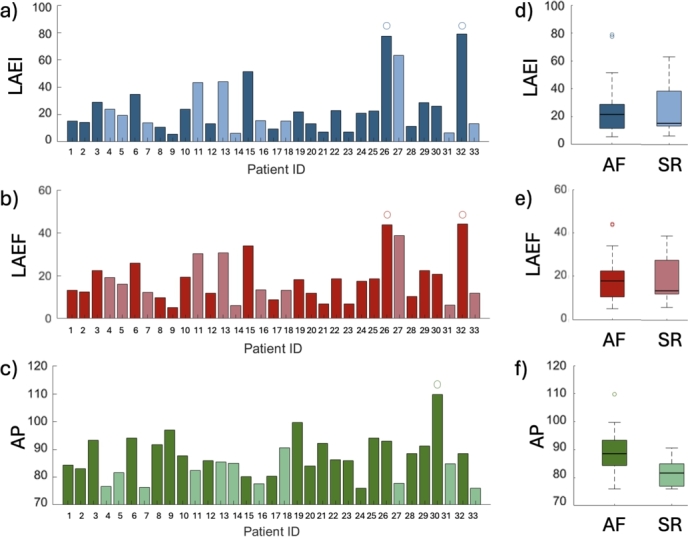
a-c) Histograms of the distribution of LAEI, LAEF, and AP features. d-f) Box plots showing the distribution of LAEI, LAEF, and AP measurements for AF and SR patients. The LAEI and LAEF are expressed as percentages, while the AP is measured in millimeters (mm). Each box plot displays the median, interquartile range, and any outliers for each parameter.

Fig. 8 illustrates the distribution of the LAEI, LAEF, and AP features for individual patients (Fig. 8 (a-c)) and the overall population of this study, divided into AF and SR group (Fig. 8 (d-f)). Panels (d) and (e) demonstrate that the dispersion of LAEI and LAEF distributions are lower in AF patients compared to those in SR. This suggests a global diminished ability of the LA to expand and contract efficiently, which is a hallmark of atrial dysfunction in AF. Panel (f) highlights that AP is larger in AF patients, due to atrial remodeling, as a consequence of AF progression. The LAEI values are presented in Fig. 8 (a, d), the LAEF values in Fig. 8 (b, e), and the AP values in Fig. 8 (c, f).

#### 3.2.2 OCSVM performance

In terms of the mean performance on the validation sets from the 5-fold cross-validation of the OCSVM algorithm, the *Sensitivity* obtained was 70%, while the *Specificity* reached 86.3%. The overall accuracy, which includes both AF and SR classes, was equal to 78.7%, demonstrating the algorithm's effective performance across both categories. Finally, the balanced accuracy, which adjusts for class imbalance, was equal to 75%.

**Fig. 9:**
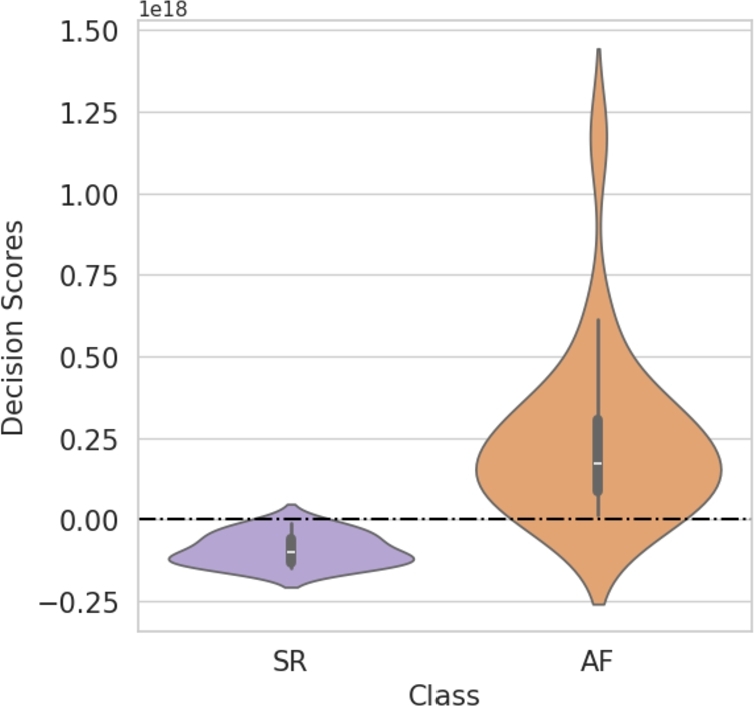
Distribution of decision function scores for the SR and AF patients computed by the OCSVM algorithm. The black dashed line indicates the primary decision boundary, corresponding to the origin of the hyperplane.

The distribution of decision function scores computed by the algorithm for both AF (inliers) and SR (outliers) is summarized in the violin diagrams depicted in Fig. 9. For the SR rhythm class, the decision scores are tightly clustered around zero with a median value of $-0.1\;\times \;10^{18}$ and variance value equal to $2.23\;\times \;10^{33}$. For the AF patients, a positively skewed distribution was obtained with a median of +0.2 and variance value equal to $6.45\;\times \;10^{34}$.

*Evaluation of the impact of volumetric features on classification -* To further evaluate the individual contribution of each feature to classification accuracy, we conducted a feature ablation analysis. Specifically, Table 5 shows the accuracy and balanced accuracy values evaluated by applying OCSVM across three feature combinations: LAEI, LAEF, and AP, and their respective pairwise combinations (LAEI+LAEF, LAEI+AP, and LAEF+AP). The highest accuracy is achieved when combining LAEI and AP (72.12%), with a similar result for LAEF+AP (72.12%). LAEI and LAEF alone perform the worst, both with an accuracy of 54.54%. In terms of balanced accuracy, LAEI+AP and LAEF+AP again yield the best performance (67.42%), while LAEI and LAEF alone show the lowest performance (45.45%).

**Table 5:** Accuracy and balanced accuracy of the OCSVM algorithm across the different features combinations.

	LAEI	LAEF	AP	LAEI+LAEF	LAEI+AP	LAEF+AP	ALL
Acc	54.54%	54.54%	66.66%	54.54%	72.12%	72.12%	78.7%
B-Acc	45.45%	45.45%	63.63%	45.45%	67.42%	67.42%	75%

#### 3.2.3 Comparison of classification methods

Table 6 presents the accuracy and balanced accuracy of the five classification methods applied to both the latent features extracted from the autoencoder (AE-DBSCAN and AE-GMM) and the original volumetric features (DBSCAN, GMM, and MS) as described in Section 2.6. Among the methods applied to the latent features, AE-GMM achieved the highest accuracy of 63.64%, outperforming AE-DBSCAN (57.57%). However, its balanced accuracy remained relatively low at 54.55%, indicating potential class imbalance effects. AE-DBSCAN, on the other hand, demonstrated the lowest balanced accuracy equal to 43.15%. Regarding the clustering performed directly on the volumetric features, DBSCAN and MS achieved the highest accuracy of 66.67%, followed closely by GMM (63.64%). Despite these improvements in accuracy, balanced accuracy remained lower, with DBSCAN and MS both at 50% and GMM slightly higher at 54.55%.

**Table 6:** Accuracy and balanced accuracy for DBSCAN and GMM applied to the latent features of the autoencoder (AE-DBSCAN and AE-GMM), and DBSCAN, GMM, and MS applied directly to the volumetric features.

	AE-DBSCAN	AE-GMM	DBSCAN	GMM	MS
Acc	57.57%	63.64%	66.67%	63.64%	66.67%
B-Acc	43.15%	54.55%	50%	54.55%	50%

## 4 Discussion and conclusions

In this study, we extended the use of 3D UNet-based segmentation, previously demonstrated on single-phase, normal ECG data, to the entire cardiac cycle in a population of AF patients. AF patients present unique technical challenges, as high heart rates, irregular rhythms, and motion artifacts, which significantly decrease image quality and complicate image segmentation. Our research demonstrates, for the first time, that a 3D-UNet-based approach can achieve accurate LA segmentation in the entire cardiac cycle in AF patients, which is crucial for the extraction of features of clinical relevance. Worth noting that, although recent approaches in the literature rely on temporal information for training AI model for automatic segmentation [59], we implemented a framework based on training with single segmentations. This design enables working with specific volumes without requiring the processing of the entire dataset and accommodates cases where not all cardiac phases have been reconstructed. This study is based on a dataset of 93 patients (60 used for the segmentation and 33 for the classification model). The study population is comparable to those in other studies based on CT images that focus on this specific region [60]. Indeed, accurate segmentation is essential for LA calculation, given the intrinsic complexity and lobate structure of its appendage. The analysis of the training trends for the Residual 3D-UNet demonstrated good convergence, with the training loss rapidly decreasing and stabilizing at approximately 0.1 after around 50 epochs (Fig. 5). The convergence of the $T_{loss}$ curve is stable and consistent across the folds, exhibiting minimal variation. Regarding the validation accuracy, the fluctuations can be attributed to the variability in the validation set or the model fine-tuning as it adapts to the data. However, also for this metric the maximum IQR was in an acceptable range (equal to 0.32). This behavior is further confirmed by validation score values for each k-fold as reported in Table 3. Additionally, all the analyzed k-fold showed a similar convergence time (Table 3). As overall consideration, the early stabilization of the training loss and high validation Dice scores demonstrated an effective model training. On the test sets, the Residual 3D-UNet demonstrated strong performance, achieving a mean Dice score of 0.94 across five-fold cross-validation, with *Pr* and *Re* also reaching 94.45% and 94.83%, respectively, as reported in Table 4. The consistent accuracy across different phases of the cardiac cycle confirms that the model can effectively handle the complex anatomical variations of the LA through the cardiac cycle. Compared to traditional manual segmentation, which is time-consuming and variable across operators, the automatic approach offers a consistent and rapid alternative, significantly reducing the time and labor burden in clinical settings. Specifically, the average time required for the manual segmentation of all 10 cardiac phases is estimated to be approximately 300 minutes per patient, whereas with the Residual 3D-UNet, the entire segmentation process is completed in about 2 minutes per patient. The model performance were assessed using an Intel(R) Core(TM) i9-10980XE CPU @ 3.00 GHz, 128 GB of RAM, and an NVIDIA GeForce RTX 3090 GPU with 24 GB of RAM. As shown in Fig. 6a, even in cases of suboptimal segmentation, the Residual 3D-UNet achieved reliable results. The qualitative and quantitative differences between manual and automatic segmentation indicated a maximum point-wise distance error of less than 2.5 mm. Moreover, this value was reached in a small area of the LA surface due to the presence of random noise in this specific region in a single phase (0%) (Fig. 6b). It is important to highlight that these discrepancies are within the range of inter- or intra-operator variability [61], [62], as reported in Fig. 6c.

Our automated pipeline enables the extraction of clinically meaningful metrics from segmented cardiac volumes, moving beyond the anatomical delineation to offer functional insights directly relevant to patient stratification and procedural planning. In particular, the framework facilitates a non-invasive, quantitative assessment of LA function across the cardiac cycle which is highly relevant for characterizing different AF phenotypes (paroxysmal, persistent, and permanent) and optimizing LAAO procedures.

Patients with AF exhibit impaired LA contractility, often reflected in reduced LAEI and LAEF values, as well as increased AP diameters [63], [64], [65]. By tracking LA volume dynamics throughout the cardiac cycle, our method captures temporal deformation patterns that allow functional discrimination between SR and AF, and potentially among AF subtypes. As shown in Fig. 7, SR patients present a typical pattern of gradual LA filling, peaking at 40% of the cardiac cycle (180 mL), followed by a marked volume reduction during atrial contraction. Conversely, AF patients (particularly those with persistent or permanent subtypes) exhibit significantly attenuated volume changes, indicating diminished mechanical activity.

From a radiomics perspective, this dynamic behavior offers quantitative markers for stratifying AF burden. For instance, the degree of LA volume variability, captured via LAEI and LAEF, may correlate with AF persistence: paroxysmal cases tend to retain partial contractile function, whereas persistent and permanent forms show minimal variation across the cycle. Thus, integrating these indices could support earlier detection and phenotyping of AF without requiring ECG data.

In the context of LAAO planning, these metrics become especially relevant. Accurate classification of AF subtype is crucial in tailoring procedural strategy, device sizing, and anticipating thromboembolic risk. Post-segmentation, our framework enables the automatic extraction of LAEI, LAEF, and AP diameters, linked to outcomes such as post-LAAO thrombus formation and recurrence of AF [66], [67], [68]. Specifically, reduced LAEF has been proposed as a marker of atrial dysfunction, which may influence patient eligibility or timing for LAAO.

As shown in Fig. 8, both individual and population-level analyses of LAEI, LAEF, and AP provide valuable insights. LAEI exhibits high inter-patient variability, with outliers above 80% particularly in early-stage or paroxysmal AF, whereas LAEF consistently clusters below 20% in persistent forms, reflecting reduced atrial output. AP diameters, centered around 85 mm, may be less discriminative alone but complement the functional indices in a multiparametric profile.

Establishing predefined threshold values for LAEI, LAEF, and AP is particularly challenging due to the nature of our study population. Unlike conventional studies that involve a binary classification of healthy and diseased individuals [69], our cohort consists entirely of patients with existing pathology. Within this group, our objective was to assess which patients exhibit a normal rhythm occurrence based on volumetric imaging features. In this context, even patients classified as SR originate from a population with underlying pathology, meaning their values are inherently altered. Consequently, there are no standardized threshold values in the literature that can serve as direct reference points under these specific conditions. Given this limitation, the OCSVM model was chosen to address the challenge of class imbalance, where AF-dominant dataset was readily available while SR cases were limited. The OCSVM model achieved an accuracy of 78.7% (Table 5), with high specificity (86.3%) in correctly identifying AF cases. While sensitivity was lower (70%), this trade-off underscores the model conservative approach, prioritizing specificity to reduce false positives, which is crucial in clinical scenarios where misclassifying AF as SR could result in undertreatment. The OCSVM algorithm demonstrates a reasonable trade-off between sensitivity and specificity, with a strong ability to detect AF while maintaining adequate detection of SR. The balanced accuracy value (75%) further supports this finding, showing that the model performs robustly across both classes without bias toward the more frequent class. Concerning the decision scores, the violin plots (Fig. 9) reveal distinct distributions of distances between the SR rhythm and the AF rhythm, with a clear separation that underscores the model ability to discriminate between the two rhythms. For the SR rhythm class (in violet), the decision scores are tightly clustered around zero, with minimal variance, indicating that these points are close to the decision boundary. Specifically, the median value is approximately −0.1, suggesting that the OCSVM model has correctly identified these instances as near-boundary points. In contrast, the AF rhythm class (in orange) displays a broader, positively skewed distribution, with the majority of distances significantly above zero and a median around +0.2. The wider spread in the inlier distribution indicates that the model effectively captures the inlier data, which are more distant from the hyperplane, suggesting higher confidence in classifying these points as inliers.

To further validate the effectiveness of the OCSVM model and justify its selection, we conducted a comparative analysis with other classification methods (Table 6). Specifically, we applied clustering techniques both to the latent features extracted from an autoencoder specifically designed to reconstruct volumetric features, and directly to the volumetric features themselves. The results suggest that while these models achieved relatively high overall accuracy, their performance across different classes was less consistent. Although AE-based clustering approaches leverage feature extraction to enhance separability, traditional clustering methods applied directly to volumetric features yielded comparable accuracy. However, the relatively low balanced accuracy values indicate challenges in maintaining consistent performance across both classes, strongly confirming that OCSVM was the most suitable approach given the nature of our dataset.

*Study limitations -* One limitation of this study is its mono-centric nature, which inherently standardizes the quality of the dataset. While this ensures consistency in imaging protocols and data acquisition, it also limits the generalizability of the AI model to broader clinical applications. Future work could address this limitation by incorporating data from multiple centers, and harmonization procedures, allowing for a more diverse dataset that captures variations in scanner technology, acquisition protocols, and patient demographics. This expansion would not only enhance the dataset size but also improve the evaluation of quality metrics, ensuring the model's robustness across different clinical settings. In addition, we will aim to enhance AI-human collaboration by improving explainability and trust in our AI models, which will be essential for their further integration into clinical practice.

Moreover, though effective, the classification model performance could be enhanced by exploring additional feature engineering or incorporating multi-modal data, such as ECG signals, echocardiography, and hemodynamic indexes [70] alongside CT-based volume metrics. Consequently, our future studies will aim to develop standardized AI algorithms capable of processing and integrating diverse imaging data while ensuring robustness, transparency, and generalizability across different patient populations. Additionally, research should focus on validating these AI models through large-scale, multicenter studies to ensure their clinical applicability and effectiveness in real-world scenarios. Furthermore, adjusting the model decision threshold or employing advanced tuning techniques might further improve sensitivity without sacrificing specificity, making the model more balanced in differentiating AF and SR. Additionally, further validation studies will be necessary to assess the model's performance in external datasets and adapt it to multi-center applications. Techniques such as domain adaptation, transfer learning, or federated learning could help mitigate the impact of inter-center variability, ensuring the AI model remains reliable across different clinical environments. Furthermore, we plan to explore avenues for improving the model's sensitivity in future iterations, which could involve refining the algorithm, incorporating additional features, or using hybrid approaches to increase its clinical applicability. Future improvements will also involve the integration of higher spatial and temporal resolution images from photon-counting computed tomography [71], which could further refine the model's ability to capture subtle structural and functional differences relevant to AF classification. Additionally, testing on an independent dataset will help assess the model's generalizability and clinical applicability. Lastly, despite the high potential of the AI-base processing tool, future improvements should include compliancies with regulatory bodies recommendations [72], [73], [74].

*Conclusion.* By enhancing segmentation accuracy and refining rhythm classification, this framework has the potential to contribute substantially to AF management, supporting clinicians in tailoring interventions to individual patients' cardiac dynamics. Additionally, the presented Residual 3D-UNet could support preoperative planning for LAAO procedures by providing precise volume measurements and shape assessment, potentially aiding in the selection of occlusion devices and enhancing patient safety by minimizing the risk of under- or over-sizing devices [75], [76]. The adoption of an AI-based segmentation algorithm has the potential to optimize treatment by reducing image processing time, increasing the reproducibility of the results thus making numerical simulations or 3D printed models a suitable tool for procedure planning within a clinical workflow. Developing our methods could bridge the gap between anatomical segmentation and clinical rhythm assessment, providing a valuable tool to enhance diagnostic accuracy and support clinical decision-making. Furthermore, as AI technology continues to evolve, further validation and integration into clinical practice will strengthen its role as a decision-support tool, improving diagnostic precision and patient outcomes in atrial fibrillation management.

## Ethics statement

The study was conducted in accordance with the Declaration of Helsinki and approved by the review board of “Comitato Etico Regionale per la Sperimentazione Clinica della Toscana-Sezione AREA VASTA NORD OVEST” (protocol code 23759, date of approval 22/02/2023).

## CRediT authorship contribution statement

**Rossana Buongiorno:** Writing – review & editing, Writing – original draft, Visualization, Validation, Software, Methodology, Investigation, Formal analysis, Data curation, Conceptualization. **Ilaria Verdirame:** Writing – review & editing, Software, Methodology, Data curation. **Francesca Dell'Agnello:** Writing – review & editing, Visualization, Software, Methodology, Formal analysis. **Benigno Marco Fanni:** Writing – review & editing, Validation, Data curation. **Katia Capellini:** Writing – review & editing, Validation, Data curation. **Alberto Clemente:** Writing – review & editing, Validation, Data curation. **Vincenzo Positano:** Writing – review & editing, Methodology. **Sergio Berti:** Writing – review & editing, Validation, Funding acquisition, Data curation. **Simona Celi:** Writing – review & editing, Writing – original draft, Visualization, Supervision, Resources, Project administration, Funding acquisition, Conceptualization.

## Declaration of Competing Interest

The authors have declared no conflict of interest.

## Figures and Tables

**Figure:**
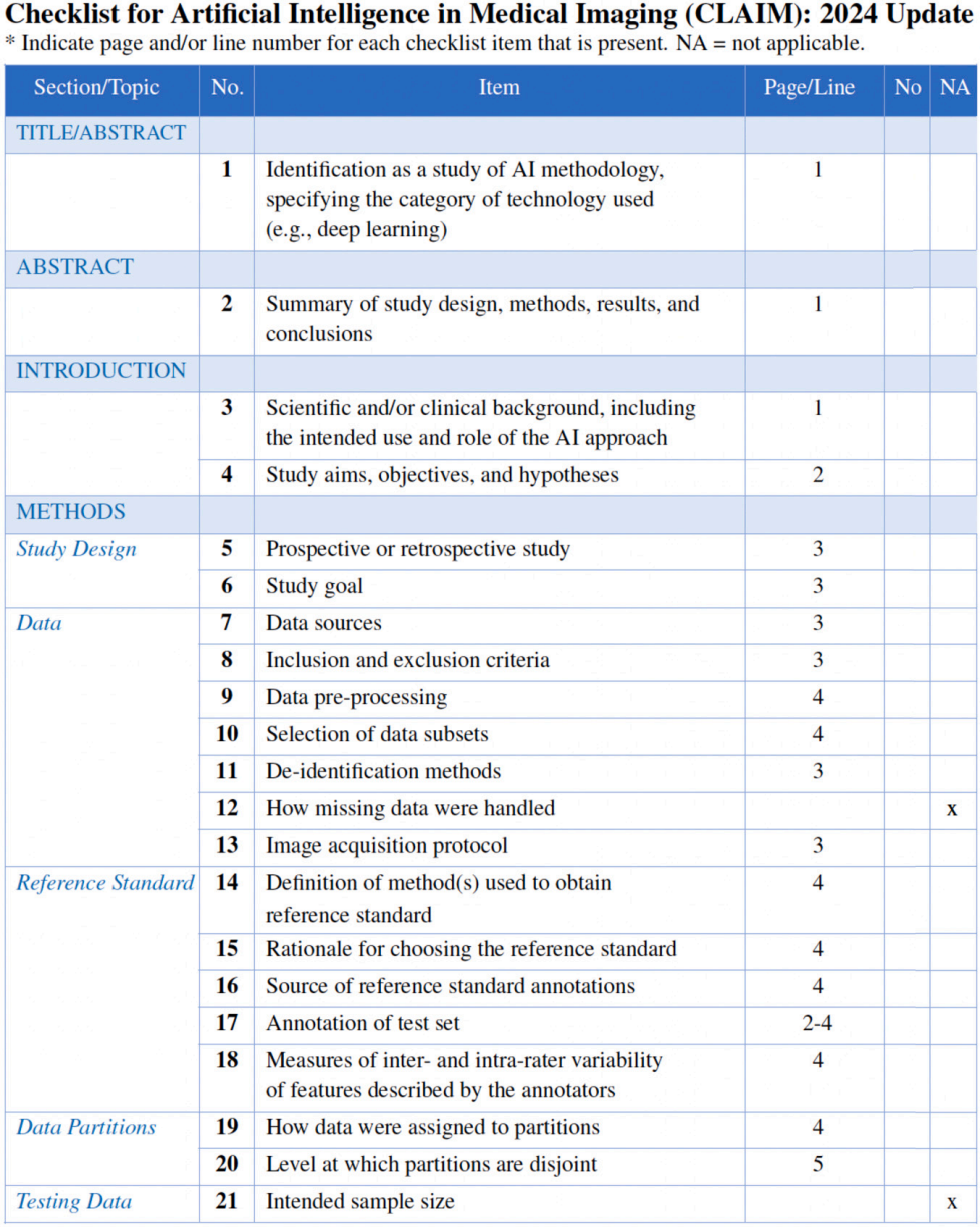


**Figure:**
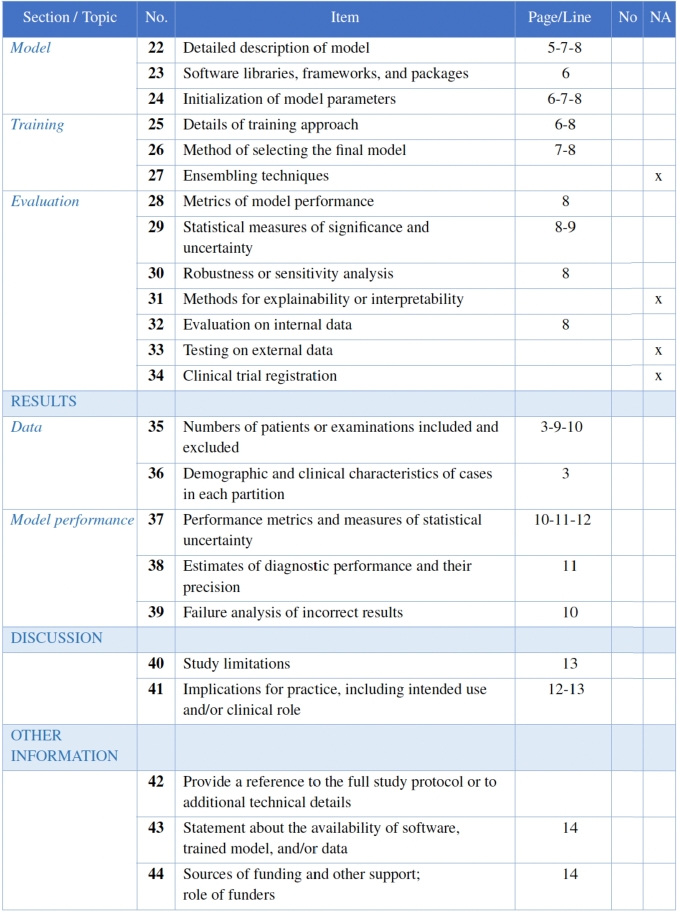


## Data Availability

To support reproducibility and future research, our full Residual 3D-UNet training and evaluation pipeline is available at https://github.com/BioEngMonasterio/segLA. Due to privacy constraints, the imaging data can be requested from the authors.

## Appendix A Checklist for artificial intelligence in medical imaging (CLAIM): 2024 update

Tejani, Ali S., et al. “Checklist for artificial intelligence in medical imaging (CLAIM): 2024 update” Radiology: Artificial Intelligence 6.4 (2024): e240300.
